# Self-Perceived Mental Health Status, Digital Activity, and Physical Distancing in the Context of Lockdown Versus Not-in-Lockdown Measures in Italy and Croatia: Cross-Sectional Study in the Early Ascending Phase of the COVID-19 Pandemic in March 2020

**DOI:** 10.3389/fpsyg.2021.621633

**Published:** 2021-02-04

**Authors:** Vanja Kopilaš, Anni M. Hasratian, Lucia Martinelli, Goran Ivkić, Lovorka Brajković, Srećko Gajović

**Affiliations:** ^1^Croatian Institute for Brain Research, School of Medicine, University of Zagreb, Zagreb, Croatia; ^2^Faculty of Croatian Studies, University of Zagreb, Zagreb, Croatia; ^3^Department of Psychology, Southern Methodist University, Dallas, TX, United States; ^4^MUSE – Science Museum, Trento, Italy

**Keywords:** pandemic (COVID-19), avoidance, intrusion, stress, depression, digital society

## Abstract

The novelty of the coronavirus disease 2019 (COVID-19) pandemic is that it is occurring in a globalized society enhanced by digital capabilities. Our aim was to analyze the psychological and emotional states of participants in different pandemic-related contexts, with a focus on their digital and physical distancing behaviors. The online survey was applied during the ascending phase of the pandemic in March 2020 in two neighboring EU countries: Italy and Croatia. The study subjects involved four groups, two directly affected by epidemiological measures and two serving as controls—(1) participants from Italy who were in lockdown (Italy group), (2) participants from Croatia who were not in lockdown but who were in direct contact with an infected person and underwent epidemiological measures (CRO-contact group), (3) participants from Croatia who were in an analogous situation but not near the same infected person (CRO-no contact group), and (4) participants from Croatia who were not aware of any infected person (CRO-unrelated group). The survey consisted of validated scales of psychological and emotional states, and custom-made questionnaires on the digital (online) and physical (off-line) behavior of the participants. The Italy group in lockdown had higher self-perceived scores for depression, stress, post-traumatic intrusion, and avoidance, as well as the highest digital activity and physical distancing than the not-in-lockdown Croatian groups. The insight into the extent of online activities and off-line isolation allowed for the introduction of Digital Activity and Physical Distancing Scores. Self-perceived post-traumatic avoidance was higher in both the Italy and CRO-contact groups than the control CRO-no contact and CRO-unrelated groups, and higher avoidance correlated with higher Digital Activity and Physical Distancing Scores. Being in direct contact with the infected person, the CRO-contact group had no other alterations than unexpectedly lower post-traumatic hyperarousal when compared with the Italy group. The Italy group in lockdown demonstrated higher self-perceived psychological toll together with higher digital activity and physical distancing than Croatian groups not in lockdown, even when compared with the affected CRO-contact group. The study outcomes suggest that the general emergency measures influenced citizens in lockdown more than exposure to the virus through direct contact with an infected person.

## Introduction

Wars, natural disasters, financial crises, terror, or similar damaging situations involving whole countries, regions, or communities affect mental and physical health and leave long-lasting personal and societal consequences. For example, wars in Afghanistan (Scholte et al., 2004) and Iraq (Taylor et al., 2014); earthquakes in Northridge, United States (McMillen et al., 2000) and L’Aquila, Italy (Ciocca et al., 2015); the 9/11 and Paris terrorist attacks (Updegraff et al., 2008; Vandentorren et al., 2018); the financial crisis during 2008 (McInerney et al., 2013); and the Ebola and swine flu pandemics (Jones and Salathé, 2009; Jalloh et al., 2018) all have one thing in common: considerable negative impact on affected communities. These unfortunate events also had notable psychological consequences, related to depression, anxiety, stress, and post-traumatic stress disorder (PTSD) (Beaglehole et al., 2018). According to the stress and coping theory, the extent of the psychological consequences depends on an energized and interchangeable relationship between individuals and their contextual environment (Lazarus and Folkman, 1984).

It is to be assumed that the coronavirus disease 2019 (COVID-19) pandemic would inflict analogous consequences on the psychological and emotional states of affected populations. However, the temporal dynamic of the pandemic, the truly global engagement, and its spread to almost every human community represent a new situation not previously encountered and therefore in need to be analyzed. The first empirical reports on psychological status of the general population due to the COVID-19 pandemic confirmed that levels of anxiety and depression were higher than those reported before the pandemic and that they increase over time (e.g., Gao et al., 2020; Wang et al., 2020). In the United States, the rate of psychological distress during the pandemic has tripled relative to the years before (McGinty et al., 2020). In another study, 35% of 52,000 participants reported psychological distress caused by the emergence of COVID-19 (Qiu et al., 2020). These findings were also demonstrated in the case of past pandemics (Hawryluck et al., 2004; Mihashi et al., 2009; Liu et al., 2012).

The additional important novelty of the current situation is that the citizens’ general habits and lifestyle have recently evolved to embrace and apply digital capabilities. By interacting and sharing the variety of contents in the digital environment, the society becomes globally connected and digitally enhanced (Svalastog et al., 2017; Kopilaš and Gajović, 2020). While feeling in danger in the off-line world, citizens currently have the alternative to operate in the online realm. Nevertheless, the digital environment is not free from risks related to mental health. A study conducted in 28 countries by Mertens et al. (2020) showed that frequent social media use and media exposure were associated with higher levels of fear that can then lead to anxiety. Misinformation is quickly distributed throughout the online realm and can cause fear, panic, and anxiety (Garfin et al., 2020). The decreased exposure to the media reports on COVID-19-related information is suggested as a protective factor against development of some psychological symptoms (Moreira and Pinto da Costa, 2020).

In this study, we were interested in the contexts surrounding individuals during the COVID-19 pandemic, and we wanted to contribute to the theoretical debate of how these contexts influenced the pandemic’s psychological consequences. As argued above, the contexts of the COVID-19 pandemic are indeed novel as they combine the global pandemic, the imposed society-wide epidemiological measures, and the globally interconnected digital society. Therefore, our aim was to examine the relation of these novel contextual aspects of the COVID-19 pandemic with psychological and emotional status of affected individuals. To reflect this aim, a cross-sectional study during the ascending phase of the pandemic in March 2020 was designed in two neighboring European countries, Croatia and Italy, to get insight into three different contextual aspects of the pandemic—being directly affected by pandemic events, following epidemiological measures, and being digitally active. The first contextual aspect relates to findings from previous pandemics (e.g., SARS) suggest that factors such as level of exposure to infection, direct contact with an infected person, and isolation have negative effects on psychological outcomes (Matsuishi et al., 2012; Lee et al., 2018). Subsequently, we examined exposure to infection by direct contact with an infected person and the general epidemiological measures of the lockdown. Moreover, we separated these two factors into two study groups, one being in direct contact with an infected person but not in lockdown, and another in lockdown but unaware of having any contact with an infected person. These two study groups were compared with the control groups who were not in lockdown or aware of contact with an infected person. Another analyzed contextual aspect was individual physical isolation and adherence to general epidemiological protective measures, and the third contextual aspect was respondents’ digital activity.

Our hypothesis was that the affected groups (individuals in direct contact with an infected person and individuals in lockdown) would report higher levels of psychological and emotional consequences compared with the control groups who were not in direct contact with an infected person or in lockdown. What was a surprising outcome was that the lockdown group reported the highest psychological disturbances, highlighting society-wide epidemiological measures as contextual contributors to the psychological consequences of the pandemic.

## Materials and Methods

### Participants and Procedure

The initiation of the study during the early ascending phase of the pandemic in March 2020 was dependent on the serendipity of having access to a unique group of Croatian Ph.D. students, who attended class (3 hours long) with an infected person identified as the third COVID-19-positive individual in Croatia. The students were notified as first-line contacts by official epidemiologists from the Croatian Institute of Public Health and were asked to avoid social gatherings for 2 weeks, to measure their temperature every day, and in case of symptoms (i.e., cough, sore throat, and fever) to stay home and call their epidemiologist. This Croatian group was henceforth referred in the study as CRO-contact group (*n* = 27). To provide adequate controls, the CRO-contact group was matched by a group of Ph.D. students at the same institution who were not in contact with the same infected person but were involved at the same time in a similar academic program (hence referred as CRO-no contact group, *n* = 21). Moreover, as an additional control, an unrelated group of students from a different institution was included, which had no knowledge of the possible COVID-19-infected students (CRO-unrelated group, *n* = 43). Finally, the study included an additional affected group, referred as Italy group (*n* = 72), which consisted of participants from Italy. Italy, at that moment in March 2020, had just entered a lockdown phase due to the ascending pandemic as the first European country that reported COVID-19 in its territory. The Italy group was recruited using convenient sampling from Trento and Bologna regions in North Italy affected by the pandemic outbreak, however, less than the Lombardy province, which was the most affected. As the whole study used email to contact the respondents, the Italy group was composed of contacts of one of the authors (LM), who were assumed to have similar education levels as the Croatian groups and being proficient in the use of the English language. Although the Croatian groups were recruited from student population (Ph.D.), these programs involve very heterogeneous attendees in regard to their age and status. Nevertheless, none of the groups were considered to be representative of the whole populations (Italians or Croatians).

A cross-sectional online study was implemented between March 4 and March 24, 2020. The online questionnaire was administered in English through the Qualtrics online survey software system (Qualtrics, Provo, United States). All participants received an invitation to participate with a link to the questionnaire sent to their email address. Participants had access to the questionnaires after they confirmed they had read the informed consent and agreed to participate in the study. In addition, all participants confirmed that they are 18 years or older and that they speak English. Participation in this study was completely voluntary, and participants did not receive any monetary compensation. The study was approved by the University of Zagreb School of Medicine Ethics Committee.

All participants, except the CRO-contact group, reported that they had not undergone testing for COVID-19, nor were they, to their knowledge, in contact with any COVID-19-infected person. A total of 231 persons were invited to participate in the online survey, and 164 individuals responded (Table 1). On the day when the participants entered the study, Italy had 31,506 infected persons and 2,503 deaths due to COVID-19, while Croatia had 10 infected persons and no deaths (Worldometers, 2020). During the period when the study groups were examined, the Italy participants were already in lockdown, whereas the Croatia participants were not. The lockdown in Croatia and the other stressful event, the March series of earthquakes (up to 5.5 M_*L*_) (Croatian Seismological Service, 2020), both occurred after the study was completed.

**TABLE 1:** Participant response rate and questionnaire answering.

	**Italy**	**CRO-contact**	**CRO-no contact**	**CRO-unrelated**	**Total**
Contacted	100	41	35	55	231
Responded	72	28	21	43	164
Response rate (Responded/contacted*100%)	72.00%	68.29%	60.00%	78.18%	71.00%
Fully completed (% of responded)	58 (81%)	18 (64%)	16 (76%)	32 (74%)	124 (76%)
Partially completed (% of responded)	14 (19%)	9 (32%)	5 (24%)	11 (26%)	39 (24%)
No-consent (% of responded)	0 (0%)	1 (4%)	0 (0%)	0 (0%)	1 (0.06%)
Outliers (% of responded)	7 (10%)	2 (7%)	2 (10%)	3 (7%)	14 (9%)

### Measures

The differences between the four analyzed groups (CRO-contact, CRO-no contact, CRO-unrelated, and Italy) were assessed by an online questionnaire that included the following subsections: demographic information (gender, age, and education), validated measures of psychological and emotional states, two sets of questions developed by the authors to assess digital and physical activities, and an open response question for additional comments.

The included validated measures covered a depth of psychological and emotional states of interest (depression, anxiety, stress, PTSD, positive and negative affect, and loneliness) and have been used in previous studies to examine people’s psychological and emotional states after stressful and potentially traumatic situations (Wang et al., 2020). The other part of the questionnaire was aimed to assess participants’ digital (online) and physical (off-line) activities during the preceding week.

#### Measures of Psychological and Emotional States

We used the English version of validated scales and questionnaires to measure psychological and emotional states. These online and self-reported tools included the following: the Depression Anxiety Stress Scale-21 (DASS-21; Lovibond and Lovibond, 1995), Impact of Event Scale-Revised (IES-R; Weiss and Marmar, 1997), the Positive and Negative Affect Schedule (PANAS; Watson et al., 1988), and the UCLA Loneliness Scale (ULS; Russell et al., 1978).

The Cronbach’s alpha was calculated to be representative of our samples. DASS-21 is a 21-item measure of self-reported symptoms of depression, anxiety, and stress over the past week. The seven-item depression scale (α = 0.92–0.96) covers hopelessness (e.g., “I felt that I had nothing to look forward to”), dysphoria (e.g., “I felt down-hearted and blue”), and anhedonia (e.g., “I couldn’t seem to experience any positive feeling at all”). The seven-item anxiety scale (α = 0.87–0.88) addresses situational anxiety (e.g., “I was worried about situations in which I might panic and make a fool of myself”) and autonomic arousal (e.g., “I experienced breathing difficulty”). The seven-item stress scale (α = 0.90–0.94) covers nervous arousal (e.g., “I felt scared without any good reason”) and difficulty relaxing (e.g., “I found it difficult to relax”). DASS-21 is rated using a 5-point scale (0 = very slightly or not at all to 4 = extremely). IES-R includes 22 items assessing subjective responses to a specific traumatic event during the past week. IES-R has three subscales: eight-item intrusion (α = 0.78–0.84) (e.g., “I had dreams about it”), eight-item avoidance (α = 0.78–0.91) (e.g., “I tried not to think about it”), and six-item hyperarousal (α = 0.71–0.90) (e.g., “I felt watchful and on-guard”). IES-R items were rated on a 0 (not at all) to 4 (extremely) scale (Weiss, 2004). PANAS is a 20-item questionnaire where 10 items measure positive affect (α = 0.87–0.92) (e.g., “Proud” “Inspired”) and 10 items measure negative affect (α = 0.82–0.93) (e.g., “Distressed” “Afraid”) over the preceding week. Participants rated the PANAS on a 1 (very slightly or not at all) to 5 (extremely) scale. ULS (α = 0.90–0.94) is a 20-item self-report measure of subjective loneliness (e.g., “I lack companionship”) and social isolation (e.g., “I am no longer close to anyone”) rated on a 4-point scale (0 = I never feel this way to 3 = I often feel this way).

#### Measures of Digital Activities

Our research team prepared 10 questions focusing on daily digital activities of participants (Supplementary Table 1). Participants rated the frequency of their daily digital activities on a 1 (very slightly or not at all) to 5 (extremely) scale. To describe the overall digital activity of the respondents by a single number, we have introduced here a novel Digital Activity Score by summing the responses on all 10 items, with higher scores indicating greater digital activity use. The Digital Activity Score demonstrated good internal consistency for this sample (α = 0.85).

#### Measures of Physical Activities

Similar to digital activities, 10 questions were prepared to explore the frequency of participants’ daily physical activities (Supplementary Table 2). Participants rated the frequency of their daily physical activities on a 1 (never) to 4 (every day) scale. Four of the 10 questions referred to activities related to individual health that may have otherwise been uncommon practices before the COVID-19 pandemic, such as “call your epidemiologist” and “measure your temperature.” Six of the 10 items referred to the extent to which individuals engaged in physical interactions with other people (e.g., go to work and spend more than 15 min in direct contact with someone). To describe the overall physical distancing of the respondents by a single number, we introduced a novel Physical Distancing Score, which was calculated by summing the six physical interaction items (Supplementary Table 2, items *a*–*f*). All items were reverse coded, with the exception of one [“Isolate yourself from others (not being in direct contact with someone)”]. A higher Physical Distancing Score indicated greater physical isolation. The six items selected for the Physical Distancing Score demonstrated adequate internal consistency for this sample (α = 0.73), whereas all 10 items of physical activities were less reliable (α = 0.44).

#### Participants’ Feelings and Experiences Related to Coronavirus Disease 2019

At the end of the questionnaire, there was an open-ended question on feelings and experiences related to the COVID-19 pandemic: “We would greatly appreciate if you would share some of your feelings and experiences related to finding out about the COVID-19 pandemic.”

### Statistical Analysis

Data were screened for outliers prior to data analysis. Data from 14 participants (CRO-contact = 2; CRO-no contact = 2; CRO-unrelated = 3; and Italy = 7) were removed because their values were three or more standard deviations from the mean on validated measures of psychological and emotional states. All data were analyzed using IBM SPSS Statistics for Macintosh (Version 23). All multiple comparisons were corrected using the Benjamini–Hochberg procedure to control for false discovery rate (Benjamini and Hochberg, 1995). Significance was set at *p* < 0.05 for all analyses.

Assumption testing was first conducted in order to use multivariate analysis of covariance (MANCOVA) to test our hypothesis of examining group differences on measures of depression, anxiety, stress, PTSD, negative and positive affect, and loneliness, controlling for age and gender. Mahalanobis distance of the nine dependent variables (DASS-21 Depression, DASS-21 Anxiety, DASS-21 Stress, IES-R Intrusion, IES-R Hyperarousal, IES-R Avoidance, PANAS Positive, PANAS Negative, and ULS) was 26.93. Therefore, multivariate normality was assumed since this was less than the critical value of the chi-square (27.88). Univariate normality assumption was violated; therefore, Pillai’s Trace test was used to interpret the MANCOVA results. Homogeneity of regression assumption was met, as all interactions between the independent variables and covariates were not significant (all *p*’s > 0.05). Pearson’s *r* correlation was used to test the assumption of no multicollinearity, and the dependent variables were moderately correlated. One-way MANCOVA was conducted with group as the independent variable, the nine scales of psychological and emotional states as the dependent variables, and age and gender as covariates. Planned contrasts were conducted to examine differences between affected (CRO-contact and Italy) and unaffected (CRO-no contact and CRO-unrelated) groups, Italy (with lockdown measures in place) and Croatia (with no lockdown measures in place), and exposed (CRO-contact) and not exposed (all other groups without contact with an infected person).

To test group differences on the frequency of digital and physical activities, we used the Kruskal–Wallis test. *Post hoc* analyses of pairwise comparisons were done by Mann–Whitney tests. One-way analysis of covariance (ANCOVA) was conducted to examine group differences on Digital Activity Scores and Physical Distancing Scores, controlling for age and gender. Pearson’s *r* correlation analyses were used to examine correlations among psychological measures, digital activities, and physical distancing.

To analyze the open-ended question, we used ATLAS.ti (ATLAS.ti Scientific Software Development GmbH, Berlin, Germany) for qualitative data analysis. The participants’ comments were coded into positive, negative, and neutral categories. ATLAS.ti keyword search feature was used to find the frequencies of the most commonly used words.

## Results

### Response Rate and Demographic Characteristics

Seventy-one percent (164/231) of participants who were contacted via email responded to the survey, of whom 75.6% (124/164) fully completed the survey (Table 1). Participants were mostly females (69.3%), and the mean age was 37.27 years (SD = 13.60) (Supplementary Table 3). Age differed significantly among the groups (*p* < 0.001). The median completion time for the study was 11.26 minutes [interquartile range (IQR) = 12.87].

### Psychological and Emotional States

In order to test if there were group differences on measures of psychological and emotional states, which included depression, anxiety, stress, PTSD, positive and negative affect, and loneliness, a one-way MANCOVA was conducted for each set of measures comparing all four groups, controlling for age and gender. The results of MANCOVA indicated that age was significantly associated with measures of psychological and emotional states [Pillai’s Trace = 0.21, *F*(9,98) = 2.82, *p* = 0.005, partial η^2^ = 0.21], but gender was not [Pillai’s Trace = 0.08, *F*(9,98) = 0.97, *p* = 0.469, partial η^2^ = 0.08]. In addition, measures of psychological and emotional states differed significantly based on group, after controlling for age and gender, Pillai’s Trace = 0.61, *F*(27,300) = 2.84, *p* < 0.001, partial η^2^ = 0.20.

**FIGURE 1:**
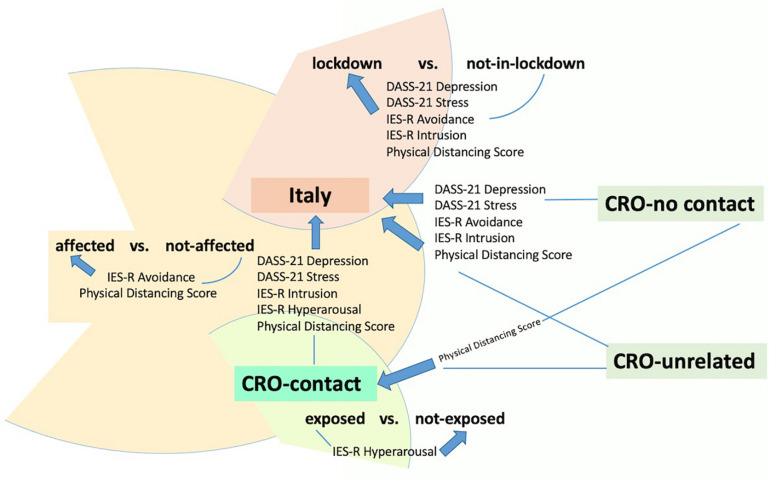
Study groups’ comparisons.

Univariate analyses revealed that younger age was associated with greater depression (*p* = 0.023) and hyperarousal (*p* = 0.003). DASS-21 Depression (*p* < 0.001), DASS-21 Stress (*p* < 0.001), IES-R Intrusion (*p* < 0.001), IES-R Hyperarousal (*p* = 0.002), and IES-R Avoidance (*p* = 0.016) were significantly different among groups (Table 2). No group differences were found on measures of anxiety, positive affect, negative affect, and loneliness. Pairwise comparisons showed that compared with the Croatian groups, the Italy group had higher DASS-21 Depression (all *p*’s ≤ 0.002) and Stress (all *p*’s ≤ 0.006) scores (Figure 1). Although the statistically significant differences among the groups were demonstrated, none of the groups’ mean scores reached levels indicative of psychopathology, all of them being well within the normal range. When impact of event was measured, the Italy group had significantly higher IES-R Intrusion than the Croatian groups (all *p*’s = 0.002), significantly higher IES-R Avoidance than the CRO-no contact and CRO-unrelated groups (both *p*’s = 0.012), and significantly higher IES-R Hyperarousal scores than CRO-contact (*p* < 0.001) (Supplementary Table 4).

**TABLE 2:** One-way MANCOVA univariate effects for group on measures of psychological and emotional states, controlling for gender and age.

**Dependent variable**	***F***	**Partial η^2^**	**Group**	**M (SD)**	**95% confidence interval**	
					**Lower bound**	**Upper bound**
DASS-21 Depression	9.19***	0.21	Italy	2.53 (2.41)	2.24	3.46
			CRO-contact	0.56 (1.55)	–0.29	1.66
			CRO-no contact	0.29 (0.73)	–0.95	1.51
			CRO-unrelated	1.31 (1.71)	–0.14	1.62
DASS-21 Anxiety	2.31	0.06	Italy	0.89 (1.28)	0.59	1.37
			CRO-contact	0.75 (1.06)	0.11	1.35
			CRO-no contact	0.14 (0.36)	–0.57	0.77
			CRO-unrelated	1.24 (1.57)	0.55	1.70
DASS-21 Stress	7.44***	0.17	Italy	3.21 (2.54)	2.89	4.30
			CRO-contact	1.44 (2.37)	0.36	2.62
			CRO-no contact	0.79 (1.58)	–0.63	1.80
			CRO-unrelated	2.14 (2.05)	0.48	2.51
IES-R Intrusion	7.16***	0.17	Italy	0.81 (0.51)	0.73	1.01
			CRO-contact	0.39 (0.35)	0.17	0.62
			CRO-no contact	0.41 (0.31)	0.14	0.62
			CRO-unrelated	0.46 (0.45)	0.16	0.57
IES-R Hyperarousal	6.12**	0.15	Italy	0.85 (0.56)	0.84	1.17
			CRO-contact	0.31 (0.35)	0.07	0.61
			CRO-no contact	0.73 (0.71)	0.36	0.94
			CRO-unrelated	1.02 (0.63)	0.51	1.00
IES-R Avoidance	4.02**	0.10	Italy	0.70 (0.56)	0.59	0.91
			CRO-contact	0.55 (0.64)	0.28	0.79
			CRO-no contact	0.29 (0.28)	–0.01	0.54
			CRO-unrelated	0.36 (0.43)	0.05	0.51
PANAS Positive	0.91	0.03	Italy	27.25 (7.50)	24.83	29.52
			CRO-contact	23.94 (7.14)	20.12	27.61
			CRO-no contact	27.07 (8.16)	23.10	31.15
			CRO-unrelated	27.24 (7.11)	24.01	30.78
PANAS Negative	2.65	0.07	Italy	17.98 (5.50)	17.31	21.07
			CRO-contact	20.75 (8.68)	18.01	24.02
			CRO-no contact	16.57 (6.27)	12.70	19.15
			CRO-unrelated	17.86 (5.57)	13.11	18.53
ULS	2.73	0.07	Italy	13.42 (8.94)	11.83	17.30
			CRO-contact	11.19 (8.98)	7.17	15.91
			CRO-no contact	8.57 (10.02)	3.25	12.64
			CRO-unrelated	17.10 (7.76)	11.83	19.07

M, mean; SD, standard deviation; DASS-21, Depression Anxiety Stress Scale-21; IES-R, Impact of Event Scale-Revised; PANAS, Positive Affect Negative Affect Schedule; ULS, UCLA Loneliness Scale; MANCOVA, multivariate analysis of covariance. **p ≤ 0.01; ***p ≤ 0.001.

#### Affected Versus Unaffected

The univariate analysis was complemented by planned contrasts for three comparisons—affected versus unaffected groups (i.e., Italy and CRO-contact vs. CRO-no contact and CRO-unrelated), lockdown versus not-in-lockdown (i.e., Italy group vs. three Croatian groups), and exposed versus not exposed (i.e., CRO-contact vs. Italy, CRO-no contact, and CRO-unrelated) (Table 3). Interestingly, while univariate analyses revealed significant group differences in the IES-R Intrusion, Hyperarousal, and Avoidance subscales, comparisons of affected groups (CRO-contact and Italy) compared with unaffected groups (CRO-no contact and CRO-unrelated) showed only a difference in IES-R Avoidance (*p* = 0.009), with the affected groups scoring higher than the unaffected groups. All other psychological measures were not significantly different between affected and unaffected groups.

**TABLE 3:** Planned contrast results for affected versus unaffected, lockdown versus not-in-lockdown, and exposed versus not exposed on measures of psychological and emotional states.

	**Affected vs. unaffected**			**Lockdown vs. not in lockdown**			**Exposed vs. not exposed**		
	**Italy CRO-contact**	**CRO-no contact CRO-unrelated**		**Italy**	**CRO-contact CRO-no contact CRO-unrelated**		**CRO-contact**	**Italy CRO-no contact CRO-unrelated**	
						
	***M* (SD)**	***M* (SD)**	***p-*value**	***M* (SD)**	***M* (SD)**	***p-*value**	***M* (SD)**	***M* (SD)**	***p-*value**
DASS-21 Depression	2.07 (2.38)	0.98 (1.54)	0.060	**2.53 (2.41)**	**0.86 (1.54)**	**<0.001**	0.56 (1.55)	1.83 (2.19)	0.268
DASS-21 Anxiety	0.86 (1.20)	0.88 (1.40)	0.641	0.89 (1.25)	0.85 (1.31)	0.607	0.75 (1.06)	0.88 (1.31)	0.981
DASS-21 Stress	2.80 (1.70)	1.70 (1.99)	0.106	**3.21 (2.54)**	**1.63 (2.08)**	**<0.001**	1.44 (2.37)	2.53 (2.42)	0.449
IES-R Intrusion	0.71 (0.51)	0.44 (0.41)	0.060	**0.81 (0.51)**	**0.43 (0.39)**	**<0.001**	0.41 (0.35)	0.64 (0.50)	0.268
IES-R Hyper-arousal	0.74 (0.56)	0.95 (0.86)	0.083	0.86 (0.56)	0.79 (0.66)	0.277	**0.37 (0.39)**	**0.90 (0.62)**	**<0.001**
IES-R Avoidance	**0.68 (0.58)**	**0.34 (0.38)**	**0.009**	**0.70 (0.56)**	**0.42 (0.48)**	**0.014**	0.63 (0.65)	0.53 (0.51)	0.386
PANAS Positive	26.48 (7.50)	27.19 (7.37)	0.444	27.25 (7.50)	26.31 (7.39)	0.607	23.94 (7.14)	27.22 (7.41)	0.268
PANAS Negative	18.62 (6.42)	17.44 (5.76)	0.246	17.98 (5.50)	18.34 (6.76)	0.739	20.75 (8.68)	17.74 (5.60)	0.302
ULS	12.89 (8.80)	14.88 (9.76)	0.743	13.26 (8.80)	13.99 (9.58)	0.739	11.68 (8.94)	13.98 (9.23)	0.596

*Significant values bolded. M, mean; SD, standard deviation; DASS-21, Depression Anxiety Stress Scale-21; IES-R, Impact of Event Scale-Revised; PANAS, positive affect negative affect schedule; ULS, UCLA Loneliness Scale.*

#### Lockdown Versus Not-in-Lockdown

The lockdown (Italy group) and not-in-lockdown (Croatian groups) contrast revealed that the Italy group had significantly higher scores in DASS-21 Depression (*p* < 0.001), DASS-21 Stress (*p* < 0.001), IES-R Avoidance (*p* = 0.014), and IES-R Intrusion (*p* < 0.001) than the three Croatian groups taken together.

#### Exposed Versus Not Exposed

The groups exposed (CRO-contact) and not exposed (Italy, CRO-no contact, and CRO-unrelated) to the virus by a direct contact with the infected person contrast showed that the CRO-contact group had significantly lower scores in IES-R Hyperarousal (*p* < 0.001) than the not exposed groups.

### Digital Activities

Frequency analyses revealed that the majority of the respondents used digital means of communication more during the week before taking the survey than compared with their previous behavior (Supplementary Table 5). This included the use of cell phones (90% used them at least moderately more than usual), computers (84%), and web browsing (86%). A quarter of the respondents declared that they used digital tools “extremely” (25% in terms of phone and computer usage and 23% for web browsing).

In relation to social media activity, 74% of respondents accessed them at least “a little,” with almost a half of them being active as producers and network creators (48% of total participants made their own posts or commented on others’ posts, and 42% added new friends). The CRO-unrelated group, being the youngest of all the groups, was a leader in passive access to the content (31% extremely scrolled through social media). Yet this was not reflected by their active content production, where they were similar to other groups (Supplementary Table 5).

The Italy group was the most pronounced in digital activities (38% extremely used computer and 31% extremely browsed the web), followed by the CRO-contact group (e.g., 22% extremely browsed the web). Furthermore, more than a fifth of the Italy group reported “extremely” in the case of actively seeking out more information on the Internet (22%) and communicating through email (21%).

Among Croatian groups, when asked specifically about browsing news websites, 22% of the CRO-contact group reported it as “extremely,” while just the opposite 25% of the CRO-unrelated group did it “very slightly or not at all.” The CRO-unrelated group in the same way “very slightly or not at all” actively sought out more information on the Internet (28%) and communicated through email (25%).

We tested if there would be group differences on frequency of digital activities. There was a statistically significant difference among groups in relation to computer use [H(3) = 18.52, *p* < 0.001], actively seeking out more information via the Internet [H(3) = 9.91, *p* = 0.019], and communication through email [H(3) = 17.13, *p* = 0.001]. Cell phone use [H(3) = 0.50, *p* = 0.92], browsing the web [H(3) = 7.78, *p* = 0.051], browsing news websites [H(3) = 6.21, *p* = 0.102], scrolling through social media [H(3) = 4.76, *p* = 0.191], adding new friends [H(3) = 7.82, *p* = 0.05], making own social media posts [H(3) = 2.06, *p* = 0.56], and commenting on other people’s posts [H(3) = 2.09, *p* = 0.553] did not significantly differ by group.

*Post hoc* pairwise comparisons revealed that there were no group differences between all Croatian groups (CRO-contact, CRO-no contact, and CRO-unrelated; all *p*’s > 0.05). The Italy group used their computers more than the CRO-unrelated (*p* < 0.001) and CRO-no contact (*p* = 0.027) groups, as well as actively sought more information via the Internet (*p* = 0.03) and communicated through email more frequently (*p* < 0.001) than the CRO-unrelated group (Supplementary Table 5).

#### Digital Activity Score

We tested if there were differences among the groups in Digital Activity Score, controlling for gender and age. A one-way ANCOVA indicated that there were no significant differences between gender [*F*(1,105) = 0.25, *p* = 0.616, partial η^2^ = 0.002], age [*F*(1,105) = 0.07, *p* = 0.790, partial η^2^ = 0.001], and groups [*F*(3,105) = 1.29, *p* = 0.282, partial η^2^ = 0.04] on Digital Activity Scores (Table 4). Moreover, planned contrasts showed no differences between affected (CRO-contact and Italy) and unaffected (CRO-no contact and CRO-unrelated) groups (*p* = 0.106), and lockdown Italy group and not-in-lockdown Croatian groups (*p* = 0.100), as well as between CRO-contact (exposed to COVID-19) and not exposed groups (*p* = 0.599) (Table 5).

**TABLE 4:** One-way ANCOVA univariate effects for group on digital activity and physical distancing scores, controlling for gender and age.

**Dependent variable**	***F***	**Partial η^2^**	**Group**	***M* (SD)**	**95% confidence interval**	
					**Lower bound**	**Upper bound**
Digital Activity Score	1.77	0.05	Italy	30.50 (7.36)	27.25	30.37
			CRO-contact	29.62 (7.47)		
			CRO-no contact	28.43 (5.89)		
			CRO-unrelated	26.69 (8.41)		
Physical Distancing Score	27.92***	0.44	Italy	14.73 (2.67)	10.17	11.43
			CRO-contact	11.81 (4.72)		
			CRO-no contact	8.29 (1.64)		
			CRO-unrelated	8.90 (2.58)		

M, mean; SD, standard deviation; ANCOVA, analysis of covariance. ***p ≤ 0.001.

**TABLE 5:** Planned contrast results for affected versus unaffected, lockdown versus not-in-lockdown, and exposed versus not exposed on digital activity score and physical distancing score.

	**Affected vs. unaffected**			**Lockdown vs. not in lockdown**			**Exposed vs. not exposed**		
	**Italy CRO-contact**	**CRO-no contact CRO-unrelated**		**Italy**	**CRO-contact CRO-no contact CRO-unrelated**		**CRO-contact**	**Italy CRO-no contact CRO-unrelated**	
						
	***M* (SD)**	***M* (SD)**	***p*-value**	***M* (SD)**	***M* (SD)**	***p*-value**	***M* (SD)**	***M* (SD)**	***p*-value**
Digital Activity Score	30.29 (6.97)	27.26 (7.66)	0.106	30.50 (6.87)	27.90 (7.62)	0.100	29.63 (7.47)	29.03 (7.38)	0.599
Physical Distancing Score	**13.65 (3.38)**	**8.70 (2.31)**	**<0.001**	**14.21 (2.67)**	**9.54 (3.41)**	**<0.001**	11.81 (4.72)	11.72 (3.72)	0.280

*Significant values bolded. M, mean; SD, standard deviation.*

### Physical Interactions

To get insight in everyday activities and compliance to epidemiological measures due to the pandemic, the custom-made questionnaire was created and analyzed (Supplementary Tables 2, 6). The two study groups were under epidemiological measures, the Italy group was in lockdown during the duration of this study, and the CRO-contact group was advised by official epidemiologists to assume self-isolation measures. Despite receiving instructions from epidemiologists, 61% of the CRO-contact respondents declared they went to work every day, and almost everybody (94%) went to the grocery store at least once (Supplementary Table 6). However, in terms of how they perceived isolation, one fifth of them (22%) declared that they actively isolated themselves, more than half of whom (67%) were still in contact with their family members.

The Italy group, which was involved in the lockdown measures, was more watchful than all Croatian groups, leading in almost all aspects of physical distancing. Quite the opposite to CRO-contact, where 61% went to work every day, 62% of the Italy group did not go to work at all. Interestingly, the Italy group exercised more than the other groups (72% exercised at least once), even though they were the oldest among the groups.

The CRO-contact group measured their temperature more frequently than others, as a recommended precaution to check if infected. The majority of participants from all groups did not consult medical professionals; however, 14 (12%) respondents did contact their physician, and four (22%) respondents from CRO-contact group contacted the epidemiologist (most likely the one who prescribed them the isolation measures).

There was a statistically significant difference between groups in going to the grocery store [H(3) = 24.09, *p* < 0.001], going to work [H(3) = 36.87, *p* < 0.001], measuring temperature [H(3) = 22.38, *p* < 0.001], calling epidemiologist [H(3) = 24.14, *p* < 0.001], visiting social gatherings [H(3) = 51.32, *p* < 0.001], spending more than 15 min in direct contact with someone [H(3) = 19.05, *p* < 0.001], and isolating self from others [H(3) = 21.06, *p* < 0.001].

Mann–Whitney *post hoc* analyses of pairwise comparisons indicated the differences between groups. The Italy group led in applying the isolation measures: they went to the grocery store fewer times, spent less time at work, and spent less time in social gatherings than each Croatian group (all *p*’s ≤ 0.008). The Italy group spent less than 15 minutes in direct contact with others more frequently than CRO-unrelated (*p* < 0.001) and isolated themselves more than CRO-no contact and CRO-unrelated (*p* < 0.001, *p* = 0.003, respectively). Interestingly, the Italy group exercised more than CRO-contact (*p* = 0.042).

Although CRO-contact group did not fully comply with the epidemiological recommendations, its members measured their temperatures more than the other three groups (all *p*’s ≤ 0.002), called their epidemiologists more than CRO-unrelated and Italy (*p* = 0.018, *p* < 0.001, respectively), and isolated themselves more than the other Croatian groups, CRO-no contact (*p* = 0.01) and CRO-unrelated (*p* = 0.037). CRO-contact spent less time in social gatherings (*p* = 0.008) and less time in direct contact with someone for 15 min or greater (*p* < 0.001) than CRO-unrelated. As expected, no group differences were found between CRO-no contact and CRO-unrelated on frequencies of physical activities (Supplementary Table 6).

#### Physical Distancing Score

When testing differences in Physical Distancing Score, the one-way ANCOVA revealed significant group differences [*F*(3,105) = 15.11, *p* < 0.001, partial η^2^ = 0.30] (Table 4). The covariates of gender [*F*(1,105) = 0.65, *p* = 0.423, partial η^2^ = 0.01] and age [*F*(1,105) = 0.17, *p* = 0.680, partial η^2^ = 0.002] were not significantly associated with Physical Distancing Score. Pairwise comparisons revealed that CRO-contact had higher Physical Distancing Score than CRO-no contact (*p* = 0.004) and CRO-unrelated (*p* = 0.013). Similarly, Italy had significantly higher Physical Distancing Score than CRO-no contact and CRO-unrelated (both *p*’s < 0.001), as well as CRO-contact (*p* = 0.013). When these two affected groups were combined in a planned contrast versus unaffected (CRO-no contact and CRO-unrelated) groups, the affected groups had a significantly higher score (*p* < 0.001). Moreover, when the Italy group in lockdown was compared with not-in-lockdown Croatian groups, they had a significantly higher score than all Croatian groups together (*p* < 0.001). No significant difference was found between the exposed (CRO-contact) and not exposed (all other) groups (*p* = 0.280) (Table 5).

### Correlation Analyses

In order to test if there was a correlation between individual behavior (digital/online and physical/off-line) and psychological and emotional states, we performed Pearson’s *r* correlation test with these parameters. To measure for digital and physical activities, we used the Digital Activity and Physical Distancing Scores introduced by this study as they were shown to have appropriate internal consistency by their α-values.

There was a significant positive correlation between the two newly introduced scores, Digital Activity and Physical Distancing Scores [*r*(122) = 0.32, *p* < 0.001], indicating that individuals who implemented more physical distancing measures increased their digital activity as well. Among the nine sets of psychological measures tested, if they correlate with Digital Activity and Physical Distancing Scores, IES-R Intrusion and Avoidance were correlated with both scores (Table 6). Higher scores on the PANAS Positive scale were positively correlated with higher engagement in digital activities (Table 6).

**TABLE 6:** Correlation between Digital Activity Score, Physical Distancing Score, and psychological and emotional states.

	**Digital activity score**	**Physical distancing score**
	***r***	***r***
DASS-21 Depression	0.09	0.23
DASS-21 Anxiety	0.20	0.15
DASS-21 Stress	0.20	0.21
IES-R Intrusion	0.32**	0.31**
IES-R Hyperarousal	0.17	0.06
IES-R Avoidance	0.30**	0.33**
PANAS Positive	0.25*	0.03
PANAS Negative	0.17	0.07
ULS	–0.16	–0.02
Digital Activity Score	−	0.30***

r, correlation; DASS-21, Depression Anxiety Stress Scale-21; IES-R, Impact of Event Scale-Revised; PANAS, Positive Affect Negative Affect Schedule; ULS, UCLA Loneliness Scale. *p < 0.05; **p < 0.01; ***p < 0.001.

### Participants’ Feelings and Experiences Related to the Coronavirus Disease 2019

Fifty-four participants provided a response to the open-ended question: “We would greatly appreciate if you would share some of your feelings and experiences related to finding out about the COVID-19 pandemic.” Frequency analyses showed that the majority of the comments were coded as negative (*n* = 30), 16 comments were coded as positive, and eight were neutral (Supplementary Table 7). The unaffected groups (CRO-no contact and CRO-unrelated) were more willing to provide responses describing their pandemic-related thoughts (60%; 29/48), of which were predominantly negative (60%, 18/30), than were the affected groups (33%; 25/76). There were no particular differences among groups apart from the unexpected outcome of the Italy group, which although showing the highest psychological scores, had the closest positive (8) to negative (9) comment ratio out of all groups.

Comment classification of negative, positive, or neutral was determined in relation to the current pandemic situation. Some respondents expressed concern and fears regarding the COVID-19 pandemic and its danger to our health. However, others expressed frustration about the COVID-19 “hysteria.”

Typical negative comments included features as in the following example: “ I am frustrated about the panic it causes and about the fact everywhere I go—someone is talking about it. I am frustrated because I feel like there are many more disasters and harmful things that are being ignored at this point, making out this virus to be the worst thing that ever happened to humanity—which is not …” (CRO-unrelated group).

An example of positive comment is: “We are a large family and have a garden. I think things are incredibly important to me now. I feel very lucky for this and I am aware that others are suffering much more than me and us. Beyond the tragedy we are experiencing, I often make considerations: slowing down was a good experience. We live in a privileged part of the world. We need to invest more in building communities. We have to learn to adapt to what is not predictable, we are not good at this, we take too many things for granted. If at the end of it all we would not have learned some important lessons, then it will not have been worth it” (Italy group).

The most common features of typical neutral comments were covered in the following example: “Every day I am getting more information about it (for instance at my work) so I am not sure what to think about COVID situation anymore” (CRO-unrelated group).

With the use of the keyword search feature, the most frequently used words used here were “people,” “panic,” “media,” “COVID,” and “worried.”

## Discussion

The uniqueness of this cross-sectional study is based on the presence of the specific groups allowing to compare tested groups with their controls and in this way introducing the elements of case–control design. The study used a very defined time window when the lockdown measures were introduced in Italy, but just before lockdown measures were subsequently introduced in Croatia. It compared the lockdown versus not-in-lockdown situation of two neighboring countries, both members of the European Union, both during the period of early rise in the numbers of infected persons. Moreover, in the affected group, CRO-contact participants were not in the lockdown (as a population-wide emergency measure); however, they were exposed as first contacts to the infected person at the same university lecture for 3 hours long and subsequently instructed with self-isolation measures by official epidemiologists. The two situations, lockdown-but-no-direct-contact and direct-contact-but-no-lockdown, were compared with the two control groups (CRO-no contact and CRO-unrelated), matched as much as the practical circumstances allowed (Figure 1).

The controls were not yet affected by the general emergency measures implemented by the state, nor by the specific measures aimed for the identified contacts of the infected persons. They served as a reference point to analyze the affected groups (CRO-contact and Italy), as they did not differ between each other in any analyzed aspects of the applied online questionnaire and showed “low profile” in the measured scores. Therefore, they were suitable to bring to light the specific changes of the affected groups. However, the control groups were not completely naïve to the pandemic. This was particularly visible in the open-ended survey question about pandemic, where the control groups were more willing to provide responses describing their thoughts, and their responses were predominantly negative.

When affected groups together were compared with the unaffected (control) groups by the set of nine psychological and emotional measures, only one feature—avoidance measured by the IES-R scale—was shown to be significantly higher in the affected groups. Avoidance of the trauma can be singled out as a key psychological consequence for the affected study groups. Due to the above-explained study design including the appropriate controls, the causality can be claimed in this relationship. Subsequently, the context of “being affected” had the consequence of psychological avoidance of the trauma.

Another unique feature of this study was the specific attention given to the digital activities being a novel online feature of the current pandemic, combined by the self-reporting of the individual physical (off-line) activity. These two contextual aspects we could report only as associations to the psychological and emotional measures, without making any conclusion about causal relationships. Interestingly, both contextual aspects were associated with each other. By measuring the digital activity (i.e., the activity in the online environment), we could demonstrate that it correlated with levels of isolation in the off-line environment. Precisely, the newly introduced Digital Activity Score and Physical Distancing Score correlated significantly with each other.

In regard to psychological and emotional states, both scores correlated with IES-R Avoidance. Subsequently, the avoidance as part of traumatic response had been identified again, but from a completely different angle as a psychological feature of the pandemic, using the digital activity and physical isolation as reference points. Moreover, we would like to suggest the triad—isolation, digital activity, and avoidance—which represents a general paradigm for dealing with the traumatic pandemic in the three spheres: off-line, online, and psychological. The avoidance measured by IES-R is shown in multiple studies to be present in traumatic situations (Peng et al., 2020; Wang et al., 2020). According to the *Diagnostic and Statistical Manual of Mental Disorders* (Fifth Edition) (DSM-5), one of the core PTSD symptoms is persistent avoidance of stimuli related to the experienced trauma (American Psychiatric Association (APA), 2013).

An important aspect of the epidemiological measures and recommendations is that citizens have no active tools against the virus, but they are offered passive isolation as a key measure of protection, avoiding the virus in a similar way as it is avoidance as a psychological feature. Presuming that both isolation and avoidance could be qualified as anti-corona measures (in both somatic and psychological sense), the increase of digital activity has allowed for compensation providing socialization while physical distancing. The study outcomes hint that dealing with the pandemic in this way can have a positive effect as the digital activities were associated with positive affect as shown by correlation to the PANAS Positive scale. In addition to IES-R Avoidance, both Digital Activity and Physical Distancing Scores significantly correlated with IES-R Intrusion as well, confirming that online digital activity and off-line isolation were associated with the traumatic consequences of the COVID-19 pandemic.

Our hypothesis that the affected groups would have higher psychological consequences than the unaffected groups was only partially confirmed, as it surprisingly turned that the two affected groups differed as well between each other. If we would discuss in general the level of likelihood to be infected, the CRO-contact group being in a direct contact with the infected person for considerable time (3 hours long), and at this moment not applying any measures of care like physical distancing or face mask wearing, could be considered to be more exposed to the virus than the Italy group, where the general emergency measures were declared by the state. However, in regard to the analyzed psychological and emotional states, the CRO-contact group did not score higher than the other groups on any of the measures surveyed. Just the opposite, the CRO-contact group surprisingly scored significantly lower on hyperarousal, as an element of traumatic response, than all other groups together. It could be concluded that the CRO-contact group did not show signs of psychological consequences due to direct contact with an infected person. More so, they were paradoxically more relaxed or less aroused regarding the traumatic influences. Suppression coping with the trauma is commonly reported when it comes to traumatic experiences (Thompson and Waltz, 2010; Mary et al., 2020). Some research even suggests that by suppressing unwanted memories, we can reduce their unconscious influence (Gagnepain et al., 2014; Wang et al., 2019).

Opposite to that, the Italy group being in lockdown, but not in direct danger of knowingly being exposed to the virus, scored higher than all other groups on four psychological scales: depression, stress, and trauma-related intrusion and avoidance. Moreover, the Italy group scored the highest in regard to digital activities and physical distancing, the latter statistically significant versus the other groups. Even when compared directly with the CRO-contact group being exposed to the virus, the Italy group scored significantly higher on measures of depression, stress, intrusion, and hyperarousal. It seems that the general emergency measures influencing the complete environment of the Italy group had a way stronger psychological effect than the individual and rather realistic danger of being in contact with the infected person (Lee and You, 2020; Xin et al., 2020). It indicated that we perceive the societal threat in a more traumatic way than the individual dangers, pointing to the importance of socialization to humans (Prime et al., 2020; Wolf et al., 2020). Another factor that could influence our findings was that at this particular moment the number of infected and deceased people was appreciably higher in Italy than in Croatia (Worldometers, 2020).

It should be noted that some measures of psychological and emotional states did not differ among the groups, UCLA scale related to loneliness, PANAS scale for both positive and negative affect, and DASS-21 Anxiety scale. Only psychological differences, but no emotional differences, were shown to differ among the study groups. This, in particular, includes no differences in loneliness, which was indicated by the previous studies to be related to depression, anxiety, and stress (Segrin and Domschke, 2011; Beutel et al., 2017; González-Sanguino et al., 2020). Isolation caused by physical distancing can lead to feelings of loneliness that can negatively impact our mental and physical health over time (Banerjee and Rai, 2020; Groarke et al., 2020). In regard to the absence of significant differences related to loneliness in our study, it could be speculated that the epidemiological isolation was compensated by digital activity.

This study reveals some practical applications worth considering in future research. The digital environment is a novelty that appears as an important contextual aspect in discussing mental health. The digital contents could be helpful in tailoring appropriate interventions, therapies, and prevention strategies in relation to the current pandemic and post-pandemic period. Future research should focus on exploration of sustainability of symptoms over time, and whether phenomena reported in our study are specific for this particular period of early pandemic or they would persist further. Adding to the contextual aspects to be elucidated, since our study was conducted in two neighboring countries, future studies could try to get insight on cross-cultural comparisons, and how cultural differences may play a role on COVID-19 effects.

### Limitations of the Study

The current study had several limitations. Although we achieved relatively high response rates, all the answers were self-reported qualifications. Due to the study design and situation specificity, the groups were rather small convenient samples, differed by age, and consisted of more females. The group differences, gender and age, were controlled in the applied statistical analyses, allowing the identification of statistically significant findings. Another limitation was the selection of English measures in non-English-speaking countries, since some of the selected measures have not been translated nor validated in Croatian and/or Italian. Although all participants confirmed proficiency in English prior to the start of the study, and the Cronbach’s alpha scores were calculated for our sample, still there may have been differences if the study was conducted in the native languages. All groups could be considered as using the English language frequently in their professional and private lives. Moreover, this allowed to administer exactly the same questionnaire to all participants, and the administration was executed to groups assumed to have appropriate knowledge of English as “lingua franca” of the current society.

Finally, the scores of all four groups were within the normal range of the validated scales. None of the groups met clinical cutoffs for certain psychological diagnoses, but they differed on the severity of normal scores. Similar to that, Wang et al. (2020) reported moderate to severe levels on psychological profiles (DASS-21 and IES-R) of their participants in the early stage of the COVID-19 pandemic.

### Conclusion

In conclusion, by using two complementary approaches (applying case–control study design and correlations of the nine dimensions of the psychological and emotional states to digital activity and physical distancing as measured by our newly introduced Digital Activity and Physical Distancing Scores), avoidance could be singled out as the major psychological consequence of the COVID-19 pandemic on the individual citizens. The observed correlations indicated that avoidance combined with increased digital activity and physical isolation would be part of the behavioral patterns during the pandemic. Moreover, the observed differences between the two affected groups indicated that the psychological response to direct, but individualized threat of infection was considerably lower than the response to collective threat represented by population-wide emergency measures. The study results could be used not only to understand the extent of the psychological toll of the pandemic on the population but as well to inform public health policies necessary to cope with the pandemic and post-pandemic challenges.

## Data Availability Statement

The raw data supporting the conclusions of this article will be made available by the authors, without undue reservation.

## Ethics Statement

The studies involving human participants were reviewed and approved by the University of Zagreb School of Medicine Ethics Committee. The participants provided their electronic informed consent to participate in this study.

## Author Contributions

VK, AH, and SG: concept and design, acquisition and interpretation of the data, and drafting the manuscript. VK, AH, LM, GI, LB, and SG: critical revision of the manuscript. AH: statistical analysis. SG: supervision. All authors have read and approved the final manuscript.

## Conflict of Interest

The authors declare that the research was conducted in the absence of any commercial or financial relationships that could be construed as a potential conflict of interest.
