# Supplementary material for: Self-Perceived Mental Health Status, Digital Activity, and Physical Distancing in the Context of Lockdown Versus Not-in-Lockdown Measures in Italy and Croatia: Cross-Sectional Study in the Early Ascending Phase of the COVID-19 Pandemic in March 2020
Source: Front Psychol. 2021 Feb 4;12:621633. doi: 10.3389/fpsyg.2021.621633 (PMC7890192; doi:10.3389/fpsyg.2021.621633)
Supplement: Supplementary file 1 [file Table_1.DOCX]

Supplementary Material

| **Table S1.** Questionnaire of the digital activity use in the past week in comparison to period before awareness of COVID-19 pandemic  *Questionnaire of the digital activity use in the past week in comparison to period before awareness of COVID-19 pandemic*  We would like to know little bit more about your digital activities.  In comparison to the period before you have been aware of the COVID-19 epidemic, IN THE PAST WEEK how often did you: | | | | | |
| --- | --- | --- | --- | --- | --- |
| a. Use your cell phone | Very slightly or not at all | A little | Moderately | Quite a bit | Extremely |
| b. Use your computer | Very slightly or not at all | A little | Moderately | Quite a bit | Extremely |
| c. Browse the web | Very slightly or not at all | A little | Moderately | Quite a bit | Extremely |
| d. Browse the news websites | Very slightly or not at all | A little | Moderately | Quite a bit | Extremely |
| e. Scroll through your social media (e.g., Facebook, Instagram) | Very slightly or not at all | A little | Moderately | Quite a bit | Extremely |
| f. Make own posts | Very slightly or not at all | A little | Moderately | Quite a bit | Extremely |
| g. Comment on other people's posts | Very slightly or not at all | A little | Moderately | Quite a bit | Extremely |
| h. Add new friends | Very slightly or not at all | A little | Moderately | Quite a bit | Extremely |
| i. Actively seek out more information via internet | Very slightly or not at all | A little | Moderately | Quite a bit | Extremely |
| j. Communicate through email | Very slightly or not at all | A little | Moderately | Quite a bit | Extremely |
