# Supplementary material for: Self-Perceived Mental Health Status, Digital Activity, and Physical Distancing in the Context of Lockdown Versus Not-in-Lockdown Measures in Italy and Croatia: Cross-Sectional Study in the Early Ascending Phase of the COVID-19 Pandemic in March 2020
Source: Front Psychol. 2021 Feb 4;12:621633. doi: 10.3389/fpsyg.2021.621633 (PMC7890192; doi:10.3389/fpsyg.2021.621633)
Supplement: Supplementary file 2 [file Table_2.DOCX]

Supplementary Material

**Table S2.** Questionnaire of the physical activity use in the past week in comparison to period before awareness of COVID-19 pandemic

| Here, we would like to know little bit more about your everyday activities.  In comparison to the period before you have been aware of the COVID-19 epidemic, IN THE PAST WEEK how often did you: | | | | | |  |
| --- | --- | --- | --- | --- | --- | --- |
| 1. Go to the grocery store | Never | Once | Few times | Every day |  | |
| 1. Visit some social gathering | Never | Once | Few times | Every day |  | |
| 1. Go to work | Never | Once | Few times | Every day |  | |
| 1. Spend more than 15 minutes in direct contact with someone | Never | Once | Few times | Every day |  | |
| 1. Being in direct contact with your family members | Never | Once | Few times | Every day |  | |
| 1. Isolate yourself from others (not being in direct contact with someone) | Never | Once | Few times | Every day |  | |
| 1. Measure your temperature | Never | Once | Few times | Every day |  | |
| 1. Exercise | Never | Once | Few times | Every day |  | |
| 1. Call your doctor | Never | Once | Few times | Every day |  | |
| 1. Call your epidemiologist | Never | Once | Few times | Every day |  | |
|  |  |  |  |  |  | |
