# Supplementary material for: Self-Perceived Mental Health Status, Digital Activity, and Physical Distancing in the Context of Lockdown Versus Not-in-Lockdown Measures in Italy and Croatia: Cross-Sectional Study in the Early Ascending Phase of the COVID-19 Pandemic in March 2020
Source: Front Psychol. 2021 Feb 4;12:621633. doi: 10.3389/fpsyg.2021.621633 (PMC7890192; doi:10.3389/fpsyg.2021.621633)
Supplement: Supplementary file 3 [file Table_3.DOCX]

Supplementary Material

| **Table S3.** Demographic characteristics of four groups | | | | | | | | | |
| --- | --- | --- | --- | --- | --- | --- | --- | --- | --- |
| Variable | Italy |  | CRO-contact |  | CRO-no contact |  | CRO-unrelated |  | Total |
| Age (year) |  |  |  |  |  |  |  |  |  |
| *M* (*SD*) | 45.92 (13.50) |  | 39.06 (6.96) |  | 32.53 (4.69) |  | 23.31 (1.18) |  | 37.27 (13.60) |
| Min-Max | 21 – 67 |  | 26 – 52 |  | 25 – 41 |  | 22 – 28 |  | 21-67 |
| Gender (% female) | 58.6% |  | 76.9% |  | 75.0% |  | 83.7% |  | 69.3% |
| *Note*. *Abbreviations*: M, Mean; SD, Standard Deviation; Min, Minimum; Max, Maximum, %, Percent. | | | | | | | | | |
