# Supplementary material for: Self-Perceived Mental Health Status, Digital Activity, and Physical Distancing in the Context of Lockdown Versus Not-in-Lockdown Measures in Italy and Croatia: Cross-Sectional Study in the Early Ascending Phase of the COVID-19 Pandemic in March 2020
Source: Front Psychol. 2021 Feb 4;12:621633. doi: 10.3389/fpsyg.2021.621633 (PMC7890192; doi:10.3389/fpsyg.2021.621633)
Supplement: Supplementary file 4 [file Table_4.DOCX]

Supplementary Material

| **Table S4.**  Pairwise comparisons for significant univariate effects for group | | | |
| --- | --- | --- | --- |
| Dependent Variable | Group | Mean | *P*-value |
|  |  |  |  |
| DASS-21 Depression | Italy  CRO-contact | 2.53  .56 | < .001 |
|  | Italy  CRO-no contact | 2.53  .29 | < .001 |
|  | Italy  CRO-unrelated | 2.53  1.31 | .002 |
|  |  |  |  |
| DASS-21 Stress | Italy  CRO-contact | 3.21  1.44 | .006 |
|  | Italy  CRO-no contact | 3.21  .79 | < .001 |
|  | Italy  CRO-unrelated | 3.21  2.14 | .006 |
|  |  |  |  |
| IES-R Intrusion | Italy  CRO-contact | .81  .39 | .002 |
|  | Italy  CRO-no contact | .81  .41 | .002 |
|  | Italy  CRO-unrelated | .81  .46 | .002 |
|  |  |  |  |
| IES-R Avoidance | Italy  CRO-no contact | .70  .29 | .012 |
|  | Italy  CRO-unrelated | .70  .35 | .012 |
|  |  |  |  |
| IES-R Hyperarousal | Italy  CRO-contact | .85  .31 | < .001 |
