# Supplementary material for: Self-Perceived Mental Health Status, Digital Activity, and Physical Distancing in the Context of Lockdown Versus Not-in-Lockdown Measures in Italy and Croatia: Cross-Sectional Study in the Early Ascending Phase of the COVID-19 Pandemic in March 2020
Source: Front Psychol. 2021 Feb 4;12:621633. doi: 10.3389/fpsyg.2021.621633 (PMC7890192; doi:10.3389/fpsyg.2021.621633)
Supplement: Supplementary file 5 [file Table_5.DOCX]

| Supplementary Material  **Table S5.**  Digital activity in the preceding week in comparison to period before awareness of COVID-19 pandemic | | | | | | | |
| --- | --- | --- | --- | --- | --- | --- | --- |
|  | **Question** | **Group** | **Ratio of respondents (n/group total)** | | | | |
|  |  |  | Very slightly or not at all | A little | Moderately | Quite a bit | Extremely |
| **MEANS OF COMMUNICATION** | Use your cell phone | Italy  CRO-contact  CRO-no contact  CRO-unrelated  Total | 0.03  0.06  0.06  0.03  0.04 | 0.05  0.06  0.06  0.06  0.06 | 0.26  0.28  0.25  0.38  0.29 | 0.41  0.33  0.50  0.22  0.36 | 0.24  0.28  0.13  0.31  0.25 |
|  | Use your computer | Italy  CRO-contact  CRO-no contact  CRO-unrelated  Total | 0.03  0.06  0.06  0.09  0.06 | 0.03  0.17  0.13  0.16  0.10 | 0.14  0.22  0.25  0.44  0.24 | 0.41  0.39  0.50  0.16  0.35 | 0.38  0.17  0.06  0.16  0.25 |
|  | Browse the web | Italy  CRO-contact  CRO-no contact  CRO-unrelated  Total | 0.02  0.06  0.06  0.06  0.04 | 0.09  0.06  0.06  0.19  0.10 | 0.21  0.44  0.38  0.34  0.30 | 0.38  0.22  0.44  0.22  0.32 | 0.31  0.22  0.06  0.19  0.23 |
|  | Browse the news websites | Italy  CRO-contact  CRO-no-contact  CRO-unrelated  Total | 0.07  0.00  0.06  0.25  0.10 | 0.10  0.17  0.06  0.16  0.12 | 0.28  0.22  0.38  0.25  0.27 | 0.38  0.39  0.44  0.19  0.34 | 0.17  0.22  0.06  0.16  0.16 |
|  | Actively seek out more information via internet | Italy  CRO-contact  CRO-no contact  CRO-unrelated  Total | 0.09  0.11  0.19  0.28  0.15 | 0.16  0.11  0.19  0.16  0.15 | 0.17  0.28  0.31  0.28  0.23 | 0.36  0.33  0.25  0.19  0.30 | 0.22  0.17  0.06  0.09  0.16 |
|  | Communicate through email | Italy  CRO-contact  CRO-no contact  CRO-unrelated  Total | 0.05  0.11  0.19  0.25  0.13 | 0.10  0.11  0.13  0.16  0.12 | 0.29  0.28  0.38  0.44  0.34 | 0.34  0.39  0.31  0.13  0.29 | 0.21  0.11  0.00  0.03  0.12 |
| **SOCIAL MEDIA** | Scroll through your social media (e.g., Facebook, Instagram) | Italy  CRO-contact  CRO-no contact  CRO-unrelated  Total | 0.19  0.11  0.06  0.06  0.13 | 0.10  0.22  0.19  0.09  0.13 | 0.22  0.33  0.44  0.28  0.28 | 0.29  0.17  0.31  0.25  0.27 | 0.19  0.17  0.00  0.31  0.19 |
|  | Make own posts | Italy  CRO-contact  CRO-no contact  CRO-unrelated  Total | 0.48  0.44  0.56  0.59  0.52 | 0.19  0.22  0.25  0.16  0.19 | 0.14  0.28  0.19  0.16  0.17 | 0.16  0.06  0.00  0.06  0.10 | 0.03  0.00  0.00  0.03  0.02 |
|  | Comment on other people's posts | Italy  CRO-contact  CRO-no contact  CRO-unrelated  Total | 0.48  0.44  0.56  0.59  0.52 | 0.22  0.28  0.19  0.22  0.23 | 0.14  0.28  0.25  0.16  0.18 | 0.14  0.00  0.00  0.03  0.07 | 0.02  0.00  0.00  0.00  0.01 |
|  | Add new friends | Italy  CRO-contact  CRO-no contact  CRO-unrelated  Total | 0.71  0.44  0.44  0.50  0.58 | 0.19  0.28  0.31  0.31  0.25 | 0.10  0.22  0.25  0.19  0.16 | 0.00  0.06  0.00  0.00  0.01 | 0.00  0.00  0.00  0.00  0.00 |
