# Supplementary material for: Self-Perceived Mental Health Status, Digital Activity, and Physical Distancing in the Context of Lockdown Versus Not-in-Lockdown Measures in Italy and Croatia: Cross-Sectional Study in the Early Ascending Phase of the COVID-19 Pandemic in March 2020
Source: Front Psychol. 2021 Feb 4;12:621633. doi: 10.3389/fpsyg.2021.621633 (PMC7890192; doi:10.3389/fpsyg.2021.621633)
Supplement: Supplementary file 6 [file Table_6.DOCX]

| ***Supplemental materials***  **Table S6.**  Physical activity use in the past week in comparison to period before awareness of COVID-19 pandemic | | | | | | |
| --- | --- | --- | --- | --- | --- | --- |
|  | **Question** | **Group** | **Ratio of respondents (n/Group total)** | | | |
|  |  |  | Never | Once | Few times | Every day |
| **EVERYDAY ACTIVITIES** | Go to the grocery store | Italy  CRO-contact  CRO-no contact  CRO-unrelated  Total | 0.21  0.06  0.06  0.03  0.12 | 0.47  0.22  0.06  0.25  0.32 | 0.28  0.39  0.69  0.69  0.45 | 0.05  0.33  0.19  0.03  0.10 |
|  | Go to work | Italy  CRO-contact  CRO-no contact  CRO-unrelated  Total | 0.62  0.28  0.13  0.22  0.40 | 0.14  0.11  0.00  0.03  0.09 | 0.12  0.00  0.06  0.09  0.09 | 0.12  0.61  0.81  0.66  0.42 |
|  | Exercise | Italy  CRO-contact  CRO-no contact  CRO-unrelated  Total | 0.28  0.56  0.38  0.41  0.36 | 0.10  0.22  0.06  0.06  0.10 | 0.45  0.17  0.31  0.47  0.40 | 0.17  0.06  0.25  0.06  0.14 |
|  | Visit some social gathering | Italy  CRO-contact  CRO-no contact  CRO-unrelated  Total | 0.81  0.50  0.25  0.09  0.51 | 0.12  0.17  0.38  0.13  0.16 | 0.05  0.22  0.31  0.66  0.27 | 0.02  0.11  0.06  0.13  0.06 |
| **COMPLIANCE WITH EPIDEMIOLOGICAL MEASURES** | Measure your temperature | Italy  CRO-contact  CRO-no contact  CRO-unrelated  Total | 0.72  0.28  0.88  0.81  0.70 | 0.10  0.22  0.13  0.09  0.12 | 0.10  0.17  0.00  0.06  0.09 | 0.07  0.33  0.00  0.03  0.09 |
|  | Call your doctor | Italy  CRO-contact  CRO-no contact  CRO-unrelated  Total | 0.90  0.78  1.00  0.88  0.89 | 0.10  0.17  0.00  0.09  0.10 | 0.00  0.06  0.00  0.03  0.02 | 0.00  0.00  0.00  0.00  0.00 |
|  | Call your epidemiologist | Italy  CRO-contact  CRO-no contact  CRO-unrelated  Total | 1.00  0.78  1.00  1.00  0.97 | 0.00  0.11  0.00  0.00  0.02 | 0.00  0.06  0.00  0.00  0.01 | 0.00  0.06  0.00  0.00  0.01 |
|  | Spend more than 15 minutes in direct contact with someone | Italy  CRO-contact  CRO-no contact  CRO-unrelated  Total | 0.33  0.11  0.00  0.00  0.17 | 0.07  0.11  0.00  0.03  0.06 | 0.09  0.39  0.25  0.09  0.15 | 0.52  0.39  0.75  0.88  0.62 |
|  | Isolate yourself from others (not being in direct contact with someone) | Italy  CRO-contact  CRO-no contact  CRO-unrelated  Total | 0.45  0.44  1.00  0.75  0.60 | 0.10  0.17  0.00  0.09  0.10 | 0.17  0.17  0.00  0.09  0.13 | 0.28  0.22  0.00  0.06  0.18 |
|  | e. Being in direct contact with your family members | Italy  CRO-contact  CRO-no contact  CRO-unrelated  Total | 0.14  0.11  0.00  0.19  0.13 | 0.03  0.00  0.00  0.03  0.02 | 0.07  0.22  0.25  0.09  0.12 | 0.76  0.67  0.75  0.69  0.73 |
