# Supplementary material for: Self-Perceived Mental Health Status, Digital Activity, and Physical Distancing in the Context of Lockdown Versus Not-in-Lockdown Measures in Italy and Croatia: Cross-Sectional Study in the Early Ascending Phase of the COVID-19 Pandemic in March 2020
Source: Front Psychol. 2021 Feb 4;12:621633. doi: 10.3389/fpsyg.2021.621633 (PMC7890192; doi:10.3389/fpsyg.2021.621633)
Supplement: Supplementary file 7 [file Table_7.DOCX]

Supplementary Material

| **Table S7.** Participants’ feelings and experiences related to the COVID-19 pandemic | | | | | |
| --- | --- | --- | --- | --- | --- |
|  | Italy | CRO-contact | CRO-no contact | CRO-unrelated | TOTAL |
| Positive | 8 | 1 | 4 | 3 | 16 |
| Negative | 9 | 3 | 4 | 14 | 30 |
| Neutral | 1 | 3 | 0 | 4 | 8 |
| TOTAL | 18 | 7 | 8 | 21 | 54 |
